# Supplementary material for: Further Evidence of the Melatonin Calmodulin Interaction: Effect on CaMKII Activity
Source: Int J Mol Sci. 2022 Feb 24;23(5):2479. doi: 10.3390/ijms23052479 (PMC8910589; doi:10.3390/ijms23052479)
Supplement: Supplementary file 1 [file ijms-23-02479-s001.zip › ijms-1551774-supplementary.pdf]

Supplementary section

UV-vis measurements set up

We analyzed the interaction between calmodulin (CaM, 4 μM) and melatonin (MEL, 1.3 μM) by UV–vis spectroscopy (figure 1s) into an i3 model spectrophotometer (Hanon Instruments Co., Ltd.) before (CaM) and after (CaM-MEL) promoting the coupling reaction.

CaM (120 μM) [1] and MEL (0.1 μM) interaction was assessed at different pH values (3.5, 4.5, 6.5 and 7.5), absorbance was measured in the wavelengths range of 250 – 320 nm (figure 2s) into an EnSpire (PerkinElmer) spectrophotometer in 96-well UV plate (Santa Cruz Biotechnology, Inc., sc-213228). The samples were measured by triplicate in independent experiments and measurements performed by spectrophotometers were done at room temperature.

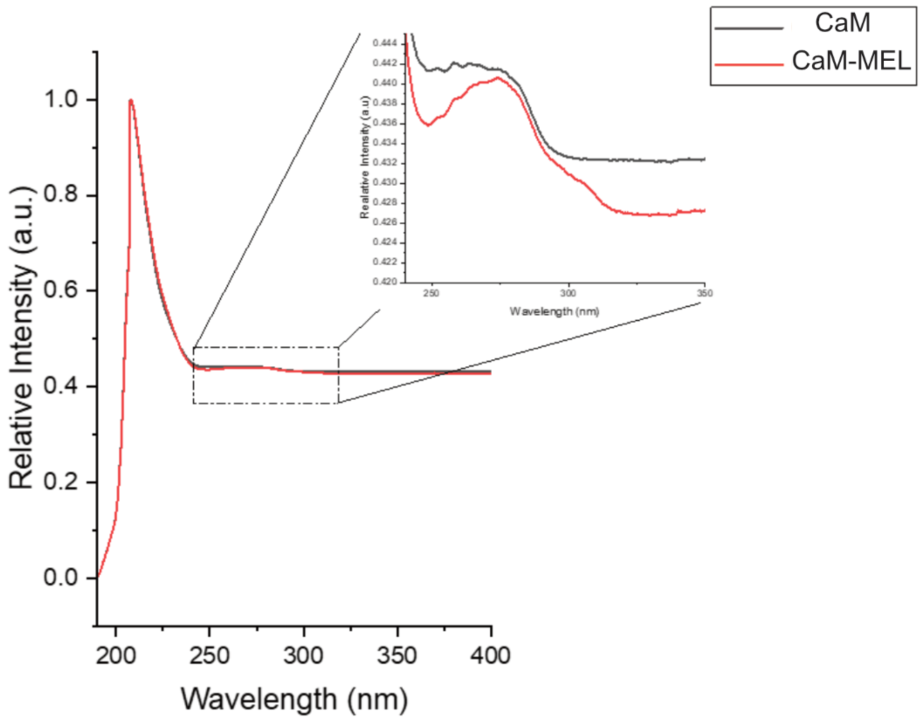

Figure S1**.** Comparison of the absorbance spectra recorded from calmodulin (CaM, 4 μM, black line) and CaM-melatonin (MEL, 1.3 μM, red line) by UV-vis spectroscopy in buffer Tris 20 mM, $MgCl_2$ 10 mM, $CaCl_2$ (0.1 mM) at pH 7.5. Note that the signals were normalized, and a representative experiment is shown.

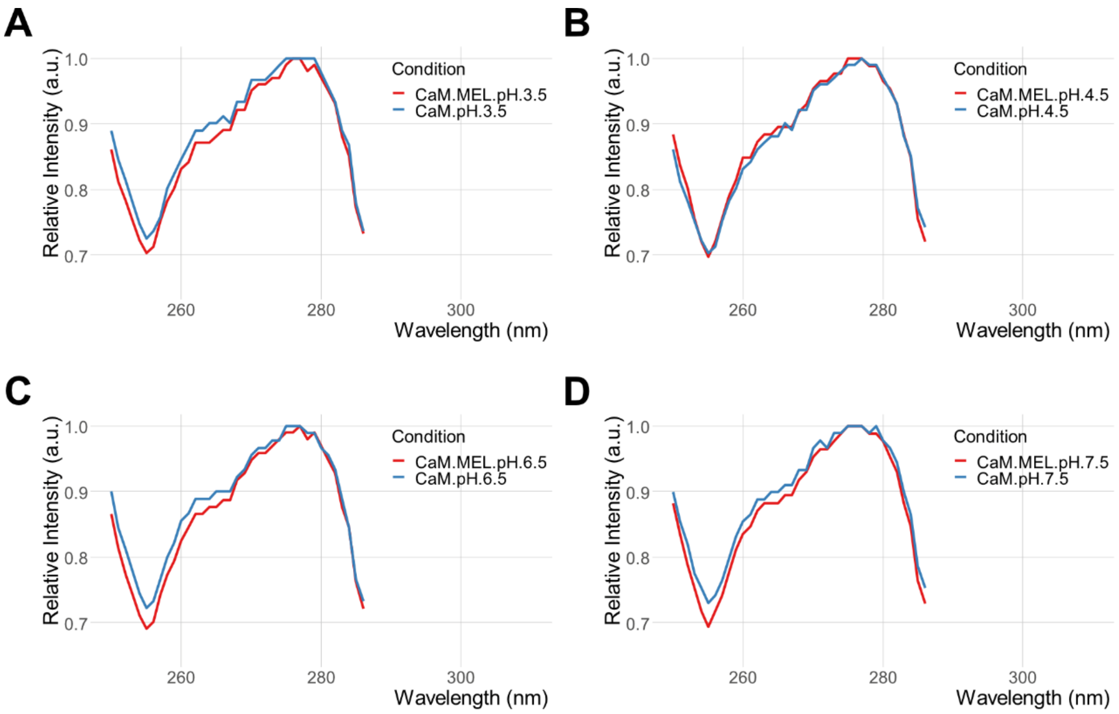

Figure S2**.** Comparison of the UV-vis absorbance spectra of Ca^{2+}-calmodulin and Ca^{2+}-calmodulin-melatonin at pH 3.5, 4.5, 6.5 and 7.5. Calmodulin (CaM, 120 μM) and CaM-melatonin (MEL, 0.1 μM) were incubated with $CaCl_2$ (0.1 mM) in Tris 20 mM and $MgCl_2$ 10 mM at pH, A) 3.5, B) 4.5, C) 6.5 and D) 7.5 at room temperature. Note that the signals were normalized, and a representative experiment is shown.

References

1. Stateva, S.R.; Salas, V.; Benaim, G.; Menéndez, M.; Solís, D.; Villalobo, A. Characterization of Phospho-(Tyrosine)-Mimetic Calmodulin Mutants. *PLOS ONE* **2015**, *10*, e0120798, doi:10.1371/JOURNAL.PONE.0120798.
